# Glucagon-like peptide-1 receptor agonists compared with bariatric metabolic surgery and the risk of obesity-related cancer: an observational, retrospective cohort study

**DOI:** 10.1016/j.eclinm.2025.103213

**Published:** 2025-05-11

**Authors:** Yael Wolff Sagy, Noga Ramot, Erez Battat, Ronen Arbel, Orna Reges, Dror Dicker, Gil Lavie

**Affiliations:** a Branch of Planning and Strategy, Clalit Health Services, Tel-Aviv, Israel; b Maximizing Health Outcomes Research Lab, Sapir College, Sderot, Israel; c Community Medical Services Division, Clalit Health Services, Tel Aviv, Israel; d Department of Health System Management, Ariel University, Ariel, Israel; e School of Medicine, Tel Aviv University, Tel Aviv, Israel; f Internal Medicine Department D and Obesity Clinic, Hasharon Hospital, Rabin Medical Center, Petah Tikva, Israel; g Ruth and Bruce Rappaport Faculty of Medicine, Technion - Israel Institute of Technology, Haifa, Israel

**Keywords:** Obesity-related cancer, Glucagon-like peptide-1 receptor (GLP1-RA), Bariatric metabolic surgery (BMS), Obesity, Diabetes

## Abstract

**Background:**

Both obesity and diabetes are associated with increased risk for specific types of cancer, generally termed obesity-related cancer (ORC). Glucagon-like peptide-1 receptor agonists (GLP-1 RAs) and bariatric metabolic surgery (BMS) are well established treatments for diabetes and obesity, but their comparative effectiveness for the prevention of ORC is unknown. Here, we compare the incidence of ORC in adults living with obesity and diabetes who were treated with first-generation GLP-1RA or with BMS.

**Methods:**

An observational, retrospective cohort study based on electronic medical records data of Clalit health services, Israel. The study included patients aged ≥24 years, with obesity and diabetes, with no prior history of ORC, who received either of the interventions during 2010 through 2018. Overall, 3178 pairs were matched 1:1 based on sex, age, baseline BMI, time of treatment initiation, and smoking status. The study period extended from January 2010 until December 2023. The measured outcome was the incidence of ORC, defined as any of the following diagnoses: multiple myeloma, meningioma, adenocarcinoma of esophagus; stomach, colorectal, liver or bile duct, gallbladder, pancreas, corpus uteri, ovary, renal-cell kidney, thyroid, and postmenopausal breast cancer.

**Findings:**

Of the 6356 study participants, 3884 (61.1%) were females. At baseline, the mean age was 52.3 years (SD: 9.28), and the mean BMI was 41.5 kg/m^2^ (SD: 5.10). Participants were followed for a median of 7.5 years and up to 12.9 years. ORC occurred in 5.62 cases per 1000 person-years in BMS patients, and in 5.89 cases per 1000 person-years in GLP-1Ra patients; adjusted HR for GLP1-RA versus BMS 1.11 (95% CI: 0.86–1.44). Moreover, assessment of mediation through weight-loss resulted in an estimated direct effect of 41% (95% CI: 15%–59%) relative risk reduction of the pharmacotherapy.

**Interpretation:**

Our finding suggest that first-generation GLP1-RA treatment does not increase ORC risk in patients receiving GLP1-RA treatment for diabetes and weight loss. Moreover, this may point at additional pathways beyond weight loss in which GLP-1RAs might contribute to the decreased risk for ORC, such as reducing inflammation. However, future studies, including randomized controlled trials and larger prospective cohort studies are needed to validate the observed effects and explore the underlying mechanisms.

**Funding:**

No financial or in-kind support was provided for the conduct of this study.

**Research in context**

**Evidence before this study**

We searched for prospective epidemiological studies and meta-analyses on the association between bariatric metabolic surgery (BMS) and obesity-related cancer (ORC), as well as on the association between treatment with glucagon-like peptide-1 receptor agonists (GLP1-RA) and ORC published in PubMed until October 27, 2024, using the search terms “bariatric” OR “GLP1-RA” OR “glucagon-like peptide-1”, AND “cancer”, AND “risk” OR “incidence”.

While BMS was demonstrated to be associated with lower incidence of ORC in a number of long-term studies and meta-analyses, evidence regarding GLP1-RA and ORC is scarce.

**Added value of this study**

This observational study of 3178 matched-pairs of patients with obesity and diabetes who received either first-generation GLP1-RAs (primarily liraglutide) or BMS, is the first to demonstrate the long-term comparative effectiveness of these treatments in adults living with obesity and diabetes with regard to the incidence of ORC (median follow-up: 7.5 years, and up to 12.9 years). The results indicate a similar incidence of ORC between the study groups, despite the relative advantage of BMS in weight reduction, known to reduce cancer risk, with a direct effect of ∼40% risk reduction of GLP1-RAs compared to BMS beyond weight-loss.

**Implications of all the available evidence**

The results reassure that GLP1-RA treatment does not increase obesity-related cancer risk when therapeutic goals beyond weight loss are considered. The estimated direct effect of GLP1-RA versus BMS on the risk for ORC may point at additional pathways beyond weight loss in which GLP-1RAs contribute to the decreased risk for ORC, such as reducing inflammation. However, future studies are needed to validate the observed effects and explore the underlying mechanisms.

## Introduction

A substantial body of evidence demonstrated the association between obesity and different types of cancer.^1^^,^^2^ In view of the magnitude of this association, in the USA obesity was considered to be the major preventable cause of cancer.^3^

The International Agency for Research on Cancer, classified specific cancer sites and types as having sufficient strength of evidence for the preventive effects of weight control on cancer risk,^1^ further termed obesity-related cancer (ORC).

Type 2 diabetes (T2DM) is also an established risk factor for certain cancers. Epidemiological studies indicated an association between T2DM and the risk for overall cancer incidence,^4^ several site-specific cancers,^5^^,^^6^ and cancer-associated mortality.^7^ Moreover, causal detrimental effects of T2DM on several cancers were demonstrated by a two-sample Mendelian randomization study.^8^

Both bariatric metabolic surgery (BMS) and treatment with GLP-1 receptor agonists (GLP-1 RAs) are offered to patients with obesity and diabetes.

BMS is well established for the treatment of severe obesity.^9^ Furthermore, BMS was demonstrated to be associated with lower incidence of ORC in a number of long-term studies and meta-analyses, with an effectiveness estimated at 32%–45%.^10^, ^11^, ^12^, ^13^, ^14^ However, less is known regarding the cancer-preventive effects of obesity pharmacotherapy.^15^ Due to the efficacy of the glucagon-like peptide 1 receptor agonist (GLP-1RA) class of pharmaceuticals in controlling T2DM, obesity, and related comorbidities, GLP1-RA might also reduce the risk for ORC. This was demonstrated in a recent comparison of patients treated with GLP1-RAs to patients who received insulin, with regard to esophageal, colorectal, endometrial, gallbladder, kidney, liver, ovarian, and pancreatic cancer, meningioma and multiple myeloma.^16^

Despite the above evidence, the comparative effectiveness of BMS and GLP1-RAs in reducing the incidence of ORC is unknown. Therefore, our objective was to compare the incidence of ORC in adults living with obesity and diabetes who were treated with BMS or first-generation GLP-1RA.

## Methods

### Study design

This observational, retrospective cohort study was based on data obtained from the electronic medical records of Clalit, the largest healthcare organization in Israel, that insures 4.78 million members (52% of the population). Clalit serves members across the entire country, with more than 1300 primary health clinics, 14 hospitals, as well as a network of medical specialists, pharmacies, and other healthcare services. In Israel, health insurance is universal and mandatory, and treatments with BMS as well as diabetes pharmacotherapies, including GLP1-RA are included in the basket of health services offered to individuals with diabetes and obesity upon clinical recommendation. Thus, all study participants were eligible for each of the measured treatments within the public health system.

Patients who underwent their first ever BMS, and patients who started treatment with GLP-1RA during the years 2010 through 2018 were matched 1:1. Exact matching was based on sex, age (in 5-years intervals), BMI (in 5-kg/m^2^ units interval), date of treatment initiation (2-years intervals), and smoking status (never smoked/past smoker/current smoker/unknown).

We defined treatment with GLP1-RA as at least 6 medication purchases within 12 months. The date of the first purchase was defined as treatment initiation. The index date was defined as 12 months after the date of BMS or initiation of GLP1-RA, in order to allow a lag-time for the assessment of the study outcome, hence avoiding immortal-time bias.

Participants who also received one of the comparative treatments (GLP1-RA patients who underwent BMS during follow-up, and patients who underwent BMS at index date and initiated treatment with GLP1-RA during follow-up), were censored from the analysis upon the initiation of the second treatment. Participants were also censored from the analysis in the event of cessation of membership in Clalit or death. Women were also censored if they were diagnosed with breast cancer before the age of 50.

The study outcome was the incidence of ORC, defined as any of the following diagnoses: multiple myeloma, meningioma, adenocarcinoma of esophagus, stomach, colorectal, liver or bile duct, gallbladder, pancreas, corpus uteri, ovary, renal-cell kidney, thyroid, and postmenopausal breast cancer.^1^

The study period extended from January 2010 until December 2023.

### Ethics

The study was approved by Clalit's Community Institutional Review Board Committee and the Clalit Health Services Data Utilization Committee, study identification number: COM2-0186-22. The Clalit's Community Institutional Review Board Committee exempted this study from the requirement to obtain informed consent owing to the analysis of anonymized patient-level data only.

### Study population

The study included Clalit members aged 24 or older, living with diabetes and obesity (BMI ≥30 kg/m^2^) who underwent BMS or initiated treatment with GLP1-RA during the study period. Patients with a history of ORC diagnosis, active cancer or end-stage renal disease during the 2 years prior the index date, or pregnancy during the year prior the index date, and patients who did not have a continous membership in Clalit for at least two years prior the index date were excluded.

Clalit's Community Institutional Review Board Committee and the Clalit Health Services Data Utilization Committee approved the study's study identification number: COM2-0186-22. The Clalit's Community Institutional Review Board Committee exempted this study from the requirement to obtain informed consent owing to the retrospective analysis of anonymized patient-level data only.

### Data extraction

We evaluated patient-level data from the operational database maintained by Clalit, which includes sociodemographic data and comprehensive clinical information, such as coexisting chronic illnesses, medications, and results of laboratory tests. Clalit pools the data from its many operational systems into a unified central data warehouse used for policy and research. This data repository includes detailed primary and secondary care information on community healthcare, medications, laboratory results, and hospitalizations. Due to the early adoption of electronic medical records and the low yearly dropout rate, Clalit has good long-term follow-up of patients, ranging from the year 2000.^17^

The following parameters at baseline were extracted from medical records for each participant: age, sex, BMI, levels of HbA1c, socioeconomic status score (SES, on a scale of 1-low to 10-high), smoking status, diagnosis of diabetes, kidney disease, history of cancer, diagnosis of alcohol use disorder, Crohn's disease, ulcerative colitis, or benign neoplasms of the colon; performance of colon screening and mammography; BMS performance by type; and purchases of GLP1-RA by type. In addition, the minimal values of BMI and HbA1c during the follow-up were extracted.

Information regarding the abovementioned chronic conditions was retrieved from the Clalit chronic diseases registry. The registry extracts information from records of primary care physicians, community specialty clinics, hospitalization discharge letters, laboratories, and pharmacies. A registry of chronic diseases diagnoses is compiled from these data sources. Diagnoses are captured in the registry by diagnosis-specific algorithms, employing International Classification of Diseases Ninth revision (ICD-9) code reading, text reading, laboratory test results and disease-specific drug usage. A record is kept of the data-sources and dates used to establish the diagnosis, with the earliest recorded date, from any source, considered to be the defining date of diagnosis.^18^

Measures of BMI were extracted from patients' community healthcare medical records. Measurements of weight (and calculated BMI) were recorded at different frequencies, upon patients' visits of primary care physicians, nurses, and dieticians. Additionally to the BMI at baseline, the lowest BMI level recorded during the follow-up period for each individual was also extracted and analysed.

All purchases of diabetes pharmacotherapies (ATC code: A10) during follow-up were extracted from pharmacy data, associated with patients' community healthcare medical records.

### Statistics

Descriptive statistics were used to characterize the study participants.

The association between treatment type and ORC was estimated as detailed. First, the following co-variables, depicting the population characteristics at baseline (beyond the matching criteria), were considered: age, SES, BMI, HbA1c levels, personal history of cancer, alcohol use disorder, Crohn's disease, ulcerative colitis, benign neoplasms of the colon, colon screening, and breast cancer screening. In the case of a high correlation between two co-variables (>0.6), only one co-variable was further analyzed. Then, univariate Cox proportional-hazards models were applied to test the associations of each of the independent candidate variable with the incidence of ORC. The criteria for inclusion of co-variables in the multivariable model was an association with the main outcome (primary incidence of ORC diagnosis), with a threshold of p < 0.25, as was previously suggested for the purposeful selection of co-variables in multivariable analyses.^19^

The reference treatment was defined as undergoing bariatric metabolic surgery (BMS). The proportional hazards model assumption was assessed using the Schoenfeld's global test. Fine and Gray competing risks regression models were used, which accounted for the effect of the competing risk of death.

Possible mediation pathways between the type of treatment and the risk for ORC were tested for changes in BMI and in HbA1c levels during the follow-up period. The potential mediators were expressed as the percent change between BMI and HbA1c values at baseline and the minimal values achieved. These changes were calculated based on the minimal value achieved during the follow-up period, while excluding the last 12 months before the end of follow-up, thus avoiding the inclusion of unintended weight-loss that may have preceded cancer diagnoses. Simple mediation models were applied. Thus, the potential mediator were each added to the multivariable Cox Proportional Hazards model. Then, the change in the main effect was assessed in order to decompose it into direct and indirect components.^20^

R statistical software version 4.0.1 (R Project for Statistical Computing) was used for the univariate and multivariate survival analysis. The following R packages were used: survival (3.2–13), ggplot2 (3.3.5), ggpubr (0.4.0), survminer (0.4.9), Table 1 (1.4.2), matchit (4.3.3), cmprsk (2.2–11), and gtsummary (1.5.2). All R packages are freely available. All reported p-values are two-tailed.

**Table 1:** Patients characteristics at baseline.

Characteristics at baseline	BMS (N = 3178)	GLP1 (N = 3178)	All participants (N = 6356)
Type of bariatric surgery, N (%)			
Sleeve gastrectomy	1550 (48.8%)		1550 (24.4%)
Gastric bypass	1280 (40.3%)		1280 (20.1%)
Laparoscopic banding	348 (11.0%)		348 (5.5%)
GLP1-RA, N (%)			
Liraglutide		2304 (72.5%)	2304 (36.2%)
Exenatide		409 (12.9%)	409 (6.4%)
Dulaglutide		357 (11.2%)	357 (5.6%)
Lixisenatide		81 (2.5%)	
Insulin Degludec and Liraglutide		25 (0.8%)	25 (0.4%)
Insulin Glargine and Lixisenatide		2 (0.1%)	2 (0.0%)
Age, years Mean (SD)	52.2 (9.26)	52.4 (9.30)	52.3 (9.28)
Female sex	1942 (61.1%)	1942 (61.1%)	3884 (61.1%)
BMI, kg/m^2^ Mean (SD)	41.7 (5.04)	41.4 (5.16)	41.5 (5.10)
HbA1c level, % Mean (SD)	6.24 (1.18)	7.69 (1.75)	6.97 (1.66)
HbA1c, missing value	22 (0.7%)	9 (0.3%)	31 (0.5%)
Personal history of cancer	213 (6.7%)	197 (6.2%)	410 (6.5%)
Socioeconimic score, Mean (SD)	5.32 (2.31)	4.51 (2.33)	4.92 (2.36)
Socioeconimic score, Median [Min, Max]	5.00 [0, 10.0]	4.00 [0, 10.0]	5.00 [0, 10.0]
Smoking status			
Never smoked	1950 (61.4%)	1950 (61.4%)	3900 (61.4%)
Current smoker	590 (18.6%)	590 (18.6%)	1180 (18.6%)
Past smoker	636 (20.0%)	636 (20.0%)	1272 (20.0%)
Unknown	2 (0.1%)	2 (0.1%)	4 (0.1%)
Alcohol use disorder	17 (0.5%)	18 (0.6%)	35 (0.6%)
Crohn's disease	9 (0.3%)	6 (0.2%)	15 (0.2%)
Colitis	11 (0.3%)	7 (0.2%)	18 (0.3%)
Benign colon neoplasms	123 (3.9%)	94 (3.0%)	217 (3.4%)
Colon cancer screening^a^			
No	641 (20.2%)	695 (21.9%)	1336 (21.0%)
Yes	1173 (36.9%)	1146 (36.1%)	2319 (36.5%)
Not eligible for screening	1364 (42.9%)	1337 (42.1%)	2701 (42.5%)
Breast cancer screening^a^			
No	241 (7.6%)	274 (8.6%)	515 (8.1%)
Yes	832 (26.2%)	827 (26.0%)	1659 (26.1%)
Not eligible for screening	2105 (66.2%)	2077 (65.4%)	4182 (65.8%)
Pharmacotherapy group (ATC codes)			
Non-insulin Glucose Lowering agents (A10BA, A10BB, A10BD, A10BG, A10BH)	2992 (94.1%)	1407 (44.3%)	4399 (69.2%)
Insulin (A10A)	2083 (65.5%)	285 (9.0%)	2368 (37.3%)
SGLT2 (A10BK)	913 (28.7%)	178 (5.6%)	1091 (17.2%)

a Eligibility for screening: Colorectal cancer: individuals aged 50–74, Breast cancer screening: women aged 50–74 years.

### Data availability

Due to Clalit Health Services' data privacy regulations and per the institutional Helsinki and data utilization committee approvals for this study, the data used for this study cannot be shared.

### Role of funding source

No financial or in-kind support was provided for the conduct of this study.

## Results

### Study population

**Fig. 1:**
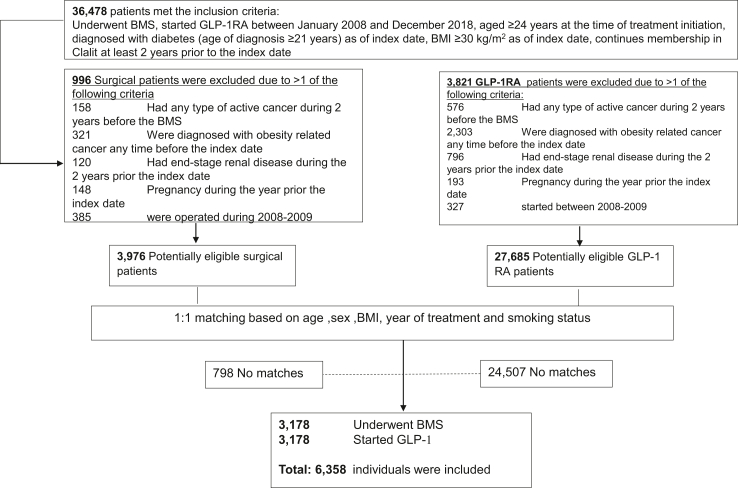
**Selection of patients who underwent BMS, and matching to patients treated with GLP-1RA.**

This observational, retrospective cohort study included Clalit Health Services members living with diabetes and obesity and with no prior history of ORC. Out of 3976 patients who underwent their first BMS, and 27,685 patient who initiated treatment with GLP1-RA during years 2010–2018 and met the study eligibility criteria, 3178 pairs were matched, with 6358 participants overall (Fig. 1). Among patients who underwent BMS at baseline, 159 had also initiated treatment with GLP1-RA during follow-up. Among patients who received GLP1-RA at baseline, 197 underwent BMS during follow-up. These patients were censored from the analysis upon the initiation of the second treatment.

Of the study participants, 3884 (61.1%) were females. At baseline, the mean age was 52.3 years (SD: 9.28), and the mean BMI was 41.5 kg/m^2^ (SD: 5.10). Of them, 410 participants (6.5%) had a history of non-obesity-related cancer. Participants were followed for a median of 7.2 years and up to 12.9 years (Table 1).

Among BMS patients, 1550 (49%) underwent sleeve gastrectomy, 1280 (40%) underwent gastric bypass, and 348 (11%) underwent laparoscopic banding.

Among GLP1-RA patients, 2304 (73%) received liraglutide, 409 (13%) received exenatide, and 357 (11%) received dulaglutide, and the remaining 4% received lixisenatide or combinations of GLP1-RAs with insulin (Table 1).

It should be noted that during the follow-up period patients could have been treated with additional diabetes medications.

### Outcomes distribution

The distribution of specific obesity-related cancer diagnoses in the study cohort is detailed in Supplementary Table S1. Among 298 patients who were diagnosed with obesity-related cancer during the follow-up period, 77 (26%) were diagnosed with breast cancer, 49 (16%) were diagnosed with malignancy of the colon or rectum, and 45 (15%) were diagnosed with malignancy of the uterus.

### Assessment of comparative treatment effectiveness

The incidence of ORC occurred in 150 of 3178 BMS patients (5.76 cases per 1000 person-years), and in 148 of 3178 GLP-1Ra patients (5.64 cases per 1000 person-years).

**Fig. 2:**
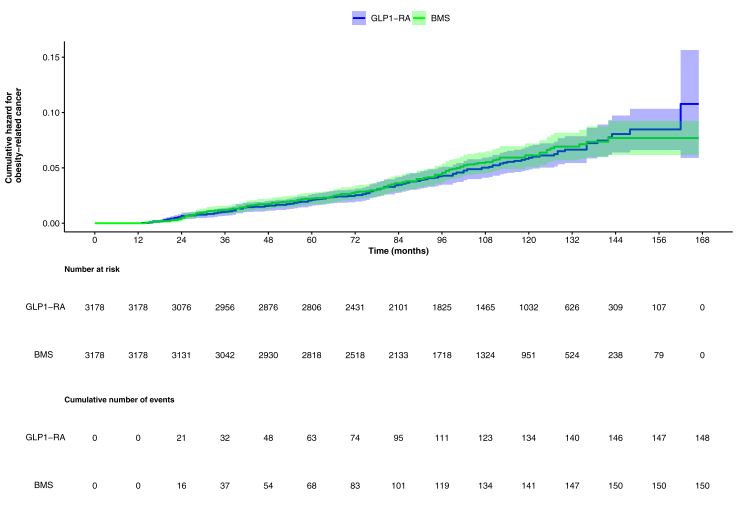
**Cumulative Hazards forobesity-relatedcancer (ORC)**. Cumulative hazards (95% CIs) for ORC are shown for patients with diabetes and obesity who underwent bariatric metabolic surgery (BMS) versus patients treated with Glucagon-Like Peptide-1 Receptor Agonists (GLP1-RA). The shaded areas represents 95% confidence intervals of the cumulative hazards.

The cumulative hazard curves are depicted in Fig. 2; adjusted HR from competing risks model for GLP1-RA versus BMS 1.03 (95% CI: 0.80–1.34) (Table 2).

**Table 2:** Association of participant characteristics and obesity-related cancer incidence.

Characteristics at baseline^a^	Univariate models		Multivariate model	
	HR (95% CI)	p value	HR (95% CI)	p value
**Treatment group**				
**BMS**	reference		reference	
**GLP1-RA**	1.07 (0.86–1.35)	0.54	0.97 (0.74–1.25)	0.79
**Age, years**	1.04 (1.03–1.05)	<0.01	1.03 (1.01–1.04)	<0.01
**BMI, kg/m^2^**	1.03 (1.00–1.05)	0.02	1.02 (0.99–1.04)	0.16
**HbA1c level, %**	0.93 (0.87–1.00)	0.06	0.95 (0.87–1.03)	0.23
**Socioeconimic score** (1, low- 10, high)	1.04 (0.99–1.09)	0.13	1.02 (0.97–1.07)	0.44
**Personal history of cancer**	0.65 (0.37–1.12)	0.12	0.46 (0.26–0.83)	0.01
**Alcohol use disorder**	0.00 (0.00–0.00)	<0.01	0.00 (0.00–0.00)	<0.01
**Crohn's disease**^b^	1.62 (0.22–11.8)	0.64	–	–
**Colitis**	2.50 (0.65–9.62)	0.18	2.59 (0.76–8.86)	0.13
**Benign colon neoplasms**	2.64 (1.73–4.03)	<0.01	2.19 (1.43–3.35)	<0.01
**Breast cancer screening**				
No	reference		reference	
Yes	1.09 (0.75–1.60)	0.64	1.12 (0.76–1.65)	0.56
Not eligible for screening	0.54 (0.37–0.78)	<0.01	0.69 (0.47–1.03)	0.07

a The variable colorectal cancer screening was not included due to high correlations (>0.6) with another variable.

b (--) variables that were not associated with the outcome in a univariate model (with a threshold of p value < 0.25) were not included in the multivariate model.

### Assessment of mediation through weight loss and HbA1c improvement

The mean percent of maximal BMI change differed between treatment groups, with a 31.1% average maximal reduction among patients who underwent BMS, and 12.9% among patients treated with first-generation GLP-1RA (Table 3), estimate from linear regression model for percent BMI change: estimate = 16.6, p < 0.001. It should be noted that these represent the lowest value during the follow-up period, and that weight-loss was not necessarily sustained. The percent maximal BMI change during follow-up was associated with the incidence of ORC (HR: 1.02, 95% CI: 1.01–1.03).

**Table 3:** Changes in BMI and HbA1c during follow-up.

	BMS (N = 3178)	GLP1 (N = 3178)	All participants (N = 6356)
**BMI**			
**Baseline BMI, kg/m^2^** mean (SD)	41.7 (5.04)	41.4 (5.16)	41.5 (5.10)
**Minimal BMI during follow-up, kg/m^2^**			
Mean (SD)	28.7 (4.85)	35.3 (5.87)	32.0 (6.31)
Missing	106 (3.3%)	164 (5.2%)	270 (4.2%)
**BMI % change (from baseline to minimal value)**			
Mean (SD)	−30.9 (10.06)	−14.3 (10.81)	−22.7 (13.3)
Missing	106 (3.3%)	164 (5.2%)	270 (4.2%)
**HbA1c levels**			
**Baseline HbA1c level, %** mean (SD)	6.24 (1.18)	7.69 (1.75)	6.97 (1.66)
**Minimal HbA1c during follow-up, mg/dL**			
Mean (SD)	5.72 (0.85)	6.82 (1.17)	6.27 (1.16)
Missing	139 (4.4%)	165 (5.2%)	304 (4.8%)
**HbA1c % change (from baseline to minimal value)**			
Mean (SD)	−8.74 (10.85)	−11.68 (14.85)	−10.21 (13.1)
Missing	139 (4.4%)	165 (5.2%)	304 (4.8%)

**Fig. 3:**
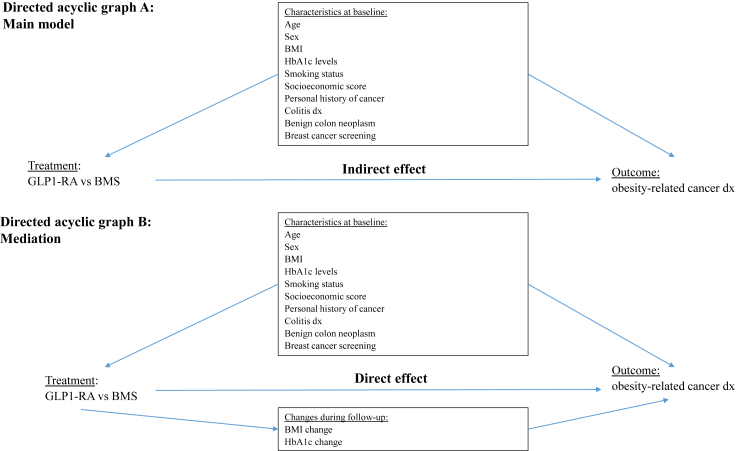
**Directed acyclic graph**. Assessing the comparative effectiveness of GLP1-RA vs BMS in obesity-related cancer. A: Main model; B: Mediation.

Adding this potential mediator to the multivariable Cox Proportional Hazards model altered the main association, adjusted HR from competing risks model for GLP1-RA versus BMS 0.59 (95% CI: 0.41–0.85). Thus, the direct effect of GLP1-RA compared to BMS on the risk for ORC beyond their effects on weight-loss is estimated as 41% (95% CI: 15%–59%) relative risk reduction of the pharmacotherapy (Fig. 3).

Similarly, a possible mediation through HbA1c change during follow-up was assessed. However, adding this potential mediator to the multivariable Cox Proportional Hazards model did not alter the main effect, adjusted HR from competing risks model for GLP1-RA versus BMS 0.87 (95% CI: 0.68–1.13) (Fig. 3).

## Discussion

In this observational, retrospective cohort study of patients living with obesity and diabetes, incidence of ORC was similar among those who were treated with first-generation GLP-1RA (primarily liraglutide) or with BMS. This is despite the relative advantage of BMS in weight reduction, known to reduce cancer risk. The results reassure that GLP1-RA treatment does not increase obesity-related cancer risk when therapeutic goals beyond weight loss are considered. Moreover, the direct effect of GLP1-RA on the risk for ORC compared to BMS beyond weight loss was estimated as 41% (95% CI: 15%–60%) relative risk reduction. No mediation though changes in HbA1c levels during the follow-up period was observed.

Similarly to our study, the comparable effect of BMS and GLP1-RA on cancer risk in individuals with BMI≥35 kg/m^2^ (not necessarily living with diabetes) was presented in a head-to-head comparison of ∼20 k propensity-score-matched pairs, based on data from TriNetX, a global healthcare database. Also, comparing each treatment to controls who received no intervention in the same study showed an advantage for GLP1-RAs.^21^

The mechanisms suggested to associate obesity with cancer risk include chronic inflammation, hormonal disorders, dysregulation of adipokines, and microbial dysbiosis.^22^ Although the mechanisms responsible for reduced cancer risk after bariatric surgery require further study, weight loss in a clear component, further strengthened by a dose-dependent response between the extent of weight loss and cancer risk among bariatric surgery patients.^23^ Furthermore, it has been suggested that BMS may attenuate excess inflammation, hyperinsulinemia, and modulate both sex hormones and adipokine levels, all associated with increased risk for different cancer types.^11^

Recent evidence suggests that GLP-1RAs may be associated with a reduced risk of certain cancers in individuals with type 2 diabetes in comparison to insulin therapy. A large retrospective cohort study by Wang L et al. demonstrated that treatment with GLP-1RAs versus insulin was associated with a lower risk for ten out of thirteen obesity-related cancers. This included colorectal (46% reduction), pancreatic (59% reduction), liver (53% reduction), gallbladder (65% reduction), kidney (24% reduction), esophageal (40% reduction), ovarian (48% reduction), and endometrial cancers (26% reduction), compared to insulin treatment.^16^ A recent drug target Mendelian randomization study suggested that GLP1-RA may decrease the risk of breast cancer and basal cell carcinoma, but increase the risk of colorectal cancer.^24^

Mechanisms associating GLP1-RAs with a reduced risk for ORC may include their contribution to weight-loss and lowering plasma glucose.^25^ However, our study results suggest a beneficial effect beyond these two pathways. Results from in-vitro and pre-clinical studies suggest anti-inflammatory properties of GLP-1RAs that may modulate the immune system. GLP-1 RAs have been associated with reductions in inflammation processes, including cardiovascular inflammation processes (notably monocyte adhesion and macrophage accumulation),^26^ neuroinflammation,^27^ and in chronic inflammatory skin disorders.^28^ Anti-inflammatory effects target different pathways in different tissues, underling a broad spectrum of GLP-1RAs actions, including the increase of anti-inflammatory cytokine level, decreasing monocytes/macrophages infiltration, and increasing regulatort T-cells.^29^ Although preliminary results are promising, it remains unclear if these will translate into long-term clinical outcomes in different patient subgroups in human. Thus, further research, including mechanistic studies is needed to ascertain possible anti-inflammatory effects of GLP1-RAs.

It should be noted that a possible association between GLP1-RAs and increased risk for cancers were reported. A pharmacovigilance study based on FDA adverse events reporting system suggested a possible association between GLP1-RAs use and increased diagnoses of pancreatic carcinoma.^30^ A systematic review and meta-analysis of randomized trials with GLP-1RAs an intervention indicated no association between the medications and pancreatic cancer, although the follow-up period was less than two years.^31^ A later analysis of the large U.S. Collaborative Network TriNetX's database reported a lower risk for pancreatic cancer among patients who had used GLP1-RAs over a follow-up period of seven years.^32^ Also, a possibly increased risk for thyroid cancer (especially medullary thyroid cancer, c-cell cancer) was suggested in view of results from pre-clinical trials, indicating an influence of GLP1-RA on increases in medullary carcinomas in rodents.^33^ However, a large cohort that followed over 145 K patients who initiated GLP1-RA treatment for ∼4 years found no increased risk for thyroid cancer incidence.^34^ For both types cancer, evidence remains inconclusive at this time, and further research is needed.^35^

The maximal BMI reduction attained during follow-up in patients who received old-generation GLP1-RAs in our study is more considerable than usually reported. This is explained by several factors, primarily the substantial multidisciplinary follow-up provided to patients, including regular dieticians' consolations. Also, the relatively high mean baseline BMI (∼41 kg/m^2^) and the assessment of the lowest weight attained during the follow-up period (that was not necessarily sustained) contribute to this result.

Our study has some noteworthy limitations. First, as in any observational study, the lack of baseline randomization implies that differences between the comparison groups may be inherent. Although robust matching for selected characteristics was applied, other differences remain, such as the baseline HbA1c levels which were lower in surgical patients. Therefore, adjustment for potential risk factors was applied, including for HbA1c levels, socioeconomic score, and clinical characteristics. Yet, residual measured or unmeasured confounders may be present, such as reasons for treatment choice including previous weight-loss failures, or surgery contraindications.

Second, the relatively low participation number is a limitation of the current study and the reproduction of similar analyses in larger cohorts is needed. However, it should be noted that cancer incidence was practically identical in the study groups, and that the adjusted point-estimate was very close to one, suggesting that the main finding of no difference between the treatment groups is not likely explained by low statistical power. In addition, the unique characteristics of the study population constrain the size of the cohort by definition, as the number of persons with obesity and diabetes who underwent bariatric metabolic surgery is relatively limited. More prospective analyses, preferably at multiple sites on a global scale are necessary to further validate the study's results before these can be translated into clinical practice implications. Also, our modest sample size did not allow to study the comparative effectiveness of treatments on the incidence of each cancer site or type separately. Rather, a composite outcome indicating the incidence of either of the thirteen ORCs was used. Previous results of GLP1-RAs effectiveness were in the same direction for 10 out of 13 cancer types,^16^ suggesting that our results can provide indication for the overall effect on ORCs.

Third, the analysis did not consider the level of adherence to GLP-1 RA treatment. Rather, an intention-to-treat approach was adopted, with a primary inclusion criteria for GLP1-RA treatment of at least 6 monthly purchases within 12 consecutive months. We have previously reported a high adherence to GLP1-RAs in a similar cohort, with a median adherence, calculated as the percent of months in which the patient had purchased GLP-1 RA during the follow-up period, of 75.6%.^36^

Fourth, in order to allow sufficiently long follow-up, our study only includes first-generation GLP1-RAs. Although the lack of newer generation GLP1-RAs is a limitation of the current study, in view of the high costs of newer generation GLP1-RAs for patients, the evaluation of first generation GLP1-RAs remains of relevance in nowadays clinical practice around the world.

A similar comparative effectiveness analysis of BMS versus new generation, highly potent GLP1-RAs may produce different results than those of the current study. This can be explored in the future with the accumulation of sufficient follow-up time.

In conclusion, this study demonstrated a similar incidence of ORC among patients treated with first-generation GLP-1RA and with BMS, despite the relative advantage of surgery in weight reduction, known to reduce the risk for ORC. The direct effect of GLP1-RA versus BMS on the risk for ORC beyond weight-loss was estimated as 41% relative risk reduction. These results may point at additional pathways in which GLP-1RAs contribute to the decreased risk for ORC, such as reducing inflammation. However, future studies, including randomized controlled trials and cohort studies on a larger global scale are needed to validate the observed effects and explore the underlying mechanisms. Furthermore, possible benefits should be weighted against safety considerations in clinical practice.

## Contributors

E.B and N.G had access to and verified the underlying data. All authors have read and agreed to the final version of the manuscript. E.B. extracted the data under the supervision of O.R. N.R. cleaned and analyzed the data with the guidance of Y.W. and O.R. N.R and Y.W drafted the initial manuscript and made the primary revisions. D.D and G.L. planned, revised, and approved all the clinical aspects of the study. R.A, Y.W and O.R planned the methodological aspects of the study. D.D, G.L, and O.R oversaw the study design and conduct.

∗Y.W and N.R contributed equally.

∗∗G.L, D.D and O.R jointly supervised this work.

## Data sharing statement

Due to Clalit Health Services' data privacy regulations and per the institutional Helsinki and data utilization committee approvals for this study, the data used for this study cannot be shared.

## Declaration of interests

Drs Dicker, Lavie, Arbel, and Reges reported receiving grants from the Israel Science Foundation outside the submitted work. Dr Dicker reported receiving grants, personal fees, and non-financial support from NovoNordisk and Eli Lilly; and personal fees and non-financial support from Boehringer Ingelheim outside the submitted work. No other disclosures were reported.
