# Supplementary material for: Glucagon-like peptide-1 receptor agonists compared with bariatric metabolic surgery and the risk of obesity-related cancer: an observational, retrospective cohort study
Source: eClinicalMedicine. 2025 May 11;83:103213. doi: 10.1016/j.eclinm.2025.103213 (PMC12208935; doi:10.1016/j.eclinm.2025.103213)
Supplement: Supplementary Table [file mmc1.docx]

## Table S1: Distribution of the cancer diagnoses during the follow-up period among study participants

| **Cancer diagnoses** | **BMS** | | **GLP1-RA** | | **All participants** | |
| --- | --- | --- | --- | --- | --- | --- |
|  | **N** | **%** | **N** | **%** | **N** | **%** |
| Postmenopausal breast cancer | 43 | 29% | 34 | 23% | 77 | 26% |
| Colorectal cancer | 24 | 16% | 25 | 17% | 49 | 16% |
| Corpus uteri cancer | 13 | 9% | 32 | 22% | 45 | 15% |
| Meningioma | 18 | 12% | 10 | 7% | 28 | 9% |
| Renal-cell kidney cancer | 18 | 12% | 8 | 5% | 26 | 9% |
| Liver or bile duct cancer | 8 | 5% | 11 | 7% | 19 | 6% |
| Pancreatic cancer | 11 | 7% | 8 | 5% | 19 | 6% |
| Thyroid cancer | 7 | 5% | 10 | 7% | 17 | 6% |
| Stomach Cancer | 3 | 2% | 5 | 3% | 7 | 2% |
| Ovarian cancer | 2 | 1% | 3 | 2% | 5 | 2% |
| Multiple myeloma | 3 | 2% | 2 | 1% | 5 | 2% |
| Overall | 150 | 100% | 148 | 100% | 298 | 100% |
